# Sport-specific training induced adaptations in postural control and their relationship with athletic performance

**DOI:** 10.3389/fnhum.2022.1007804

**Published:** 2023-01-12

**Authors:** Erika Zemková, Zuzana Kováčiková

**Affiliations:** ^1^Department of Biological and Medical Sciences, Faculty of Physical Education and Sport, Comenius University in Bratislava, Bratislava, Slovakia; ^2^Faculty of Health Sciences, University of St. Cyril and Methodius in Trnava, Trnava, Slovakia; ^3^Institute of Physical Education and Sport, Pavol Jozef Šafárik University in Košice, Košice, Slovakia

**Keywords:** athletes, neuromuscular functions, performance measures, postural sway variables, static and dynamic balance

## Abstract

Effects of various exercise programs on postural balance control in athletes and their underlying physiological mechanisms have been extensively investigated. However, little is known regarding how challenging sport-specific conditions contribute to the improvement of body balance and to what extent these changes may be explained by sensorimotor and/or neuromuscular function adaptations. Analysis of the literature could provide useful information on the interpretation of changes in postural sway variables in response to long-term sport-specific training and their association with performance measures. Therefore, the aim of this scoping review was (1) to analyze the literature investigating postural control adaptations induced by sport-specific training and their relationship with measures of athletic performance, and (2) to identify gaps in the existing research and to propose suggestions for future studies. A literature search conducted with Scopus, Web of Science, MEDLINE and Cochrane Library was completed by Elsevier, SpringerLink and Google Scholar with no date restrictions. Overall, 126 articles were eligible for inclusion. However, the association between variables of postural balance control and measures of sport-specific performance was investigated in only 14 of the articles. A relationship between static and/or dynamic balance and criterion measures of athletic performance was revealed in shooting, archery, golf, baseball, ice-hockey, tennis, and snowboarding. This may be ascribed to improved ability of athletes to perform postural adjustments in highly balanced task demands. However, the extent to which sport-specific exercises contribute to their superior postural stability is unknown. Although there is a good deal of evidence supporting neurophysiological adaptations in postural balance control induced by body conditioning exercises, little effort has been made to explain balance adaptations induced by sport-specific exercises and their effects on athletic performance. While an enhancement in athletic performance is often attributed to an improvement of neuromuscular functions induced by sport-specific balance exercises, it can be equally well ascribed to their improvement by general body conditioning exercises. Therefore, the relevant experiments have yet to be conducted to investigate the relative contributions of each of these exercises to improving athletic performance.

## 1. Introduction

Long-term adaptations in postural balance control following a variety of exercises have been extensively researched (Behm et al., 2015; Hammami et al., 2016; Granacher and Behm, 2022). In particular, balance exercises lead to neurophysiological adaptations, which is beneficial for improvement of physical performance including posture, strength and jumping (Taube et al., 2008). The enhancement in motor skills following balance training can often be observed in increasing the rate of force development (Hrysomallis, 2011). Supraspinal adaptations within the central nervous system (CNS) are mainly responsible for improving functional parameters like balance skills, explosive strength or coordinative movement control (Taube, 2012). This suggests plasticity of the sensorimotor system, particularly the spinal and supraspinal structures (Taube et al., 2008). This plasticity of the spinal, corticospinal and cortical pathways is highly task specific (Taube et al., 2008).

Postural adaptation may be ascribed to improved ability to regulate the center of mass (CoM) movement more precisely with less effort and to perceive movement of the CoM more accurately via the use of proprioceptors. This is mostly true for task-oriented balance exercises based on visual feedback control of the CoM position (Zemková, 2014a). Although it is not possible to separate sensory and motor components of balance ability, practice can improve mainly proprioceptive functions. This is because the same receptors contribute to discriminating the position of ankle joints while regulating postural sway in the anteroposterior direction and transmissing the weight from one leg to the other while regulating postural sway in the mediolateral direction. This contributes to our understanding of physiological mechanisms underlying improvements of postural stability after body conditioning exercises (Zemková, 2010).

However, research to date has only marginally addressed their relevance with respect to performance in sports where static and/or dynamic balance plays an essential role. Postural adaptations are specific to the context in which the physical activity is practiced, so there is only slight transfer to non-experienced motor tasks (Paillard, 2017). However, the adaptation may occur as part of the interlimb relationship, particularly when the two legs do not display the same motor experience (Paillard, 2017). The most successful competitive athletes have more elaborate postural strategies compared with athletes at lower competition level (Paillard, 2017). They have the best postural performance both in ecological (specific postural conditions related to the sport practiced) and non-ecological (decontextualized postural conditions in relation to the sport practiced) postural conditions (Paillard, 2017). However, in non-ecological conditions, the postural tasks should be preferentially challenging or relatively close to the sport practice stance (Paillard, 2017). Though balance training improves performance of sport-related and postural control measures, it is unclear whether the effect of training would transfer to general functional enhancement (Yaggie and Campbell, 2006).

A novel approach is necessary to provide the basis for transfer of underlying sensorimotor processes of postural control to specific sport environments. There is a need to analyze the existing literature and elucidate whether the environment in which exercises are performed plays a role in an improvement of postural stability relevant to sport-specific skills. Provided that body balance is a complex adaptive system interacting with the environment in a functionally integrated manner, the interrelation between the sensorimotor processes of postural control and sport-specific tasks may be assumed. What is lacking is a review of the evidence investigating how these components are modified with training and subsequently how they influence performance in sports with high demands on body balance.

Therefore, the aim of this scoping review was to analyze literature investigating postural control adaptations induced by sport-specific training and their relationship with measures of athletic performance. This provided a basis for identifying gaps in the existing research and suggesting recommendations for future studies.

## 2. Methods

This paper is presented in a form of a scoping review (Arksey and O’Malley, 2005; Armstrong et al., 2011; Sucharew and Macaluso, 2019). It addresses two research questions: Do sport-specific balance exercises contribute to the enhancement of athletic performance in sports with high demands on postural stability? Is there a connection between improvements in postural control induced by sport-specific training and athletic performance?

A literature review was made to analyze existing research related to the relationship between variables of postural balance control and athletic performance measures, and related neurophysiological adaptations induced by sport-specific balance exercises. The search conducted with Scopus, Web of Science, MEDLINE and Cochrane Library was completed by Elsevier, SpringerLink and Google Scholar with no date restrictions. Articles in peer-reviewed journals were analyzed. However, references included in reviews were also manually searched to identify other relevant studies. If overlapping data were found in multiple articles, resulting from similar or the same research, those with the most recent publication date were considered for analysis. Books, theses, case reports, abstracts or articles published in conference proceedings were excluded. Incomplete articles and studies that did not include original research were also excluded. The inclusion criteria included research papers that sufficiently described participants, study design, and relevant measures. Studies written in English were preferred. Papers that failed to meet the eligibility criteria were excluded.

The search was focused on studies close to the main aim of this review. The key inclusion criterion was that (a) the training included specific balance exercises performed within a given sport, (b) variables of postural balance control were related to athletic performance measures, and (c) postural adaptations induced by sport-specific training were analyzed. However, only a small number of studies was revealed using this approach. Therefore, the search was widened to studies investigating adaptations in postural balance control and athletic performance induced by sport-specific as well as general body conditioning exercises. This helped us to identify gaps in the existing research and propose recommendations for further studies on this topic.

The search and appraisal of studies selected by inclusion and exclusion criteria was conducted by both authors. Some concerns were about the representativeness of samples, missing information related to balance exercises included in sport-specific training programs, imprecisely described variables of postural balance control and related performance measures and/or non-controlled compliance of experiments. Athletes of individual and team sports where postural stability plays an important role in their performance were considered a target population.

**FIGURE 1:**
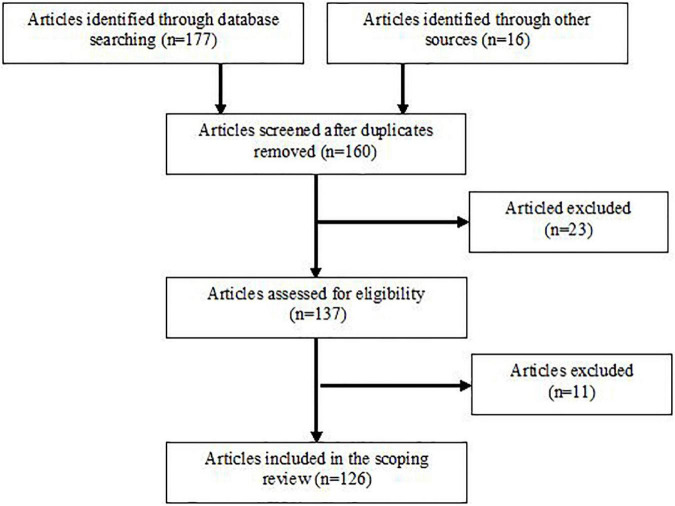
Flow chart illustrating phases of the literature search and study selection.

In the search strategy were included suggested sports combined with these terms: “balance exercises” AND “sport-specific exercise” AND “balance” AND “postural control” AND “athletes” AND “athletic performance” AND “postural sway variables” AND “performance measures” AND “neurophysiological adaptations,” AND “neuromuscular functions,” AND “sensorimotor functions,” AND “physiological mechanisms.” Further searches were performed using words from subheadings that specified other balance related exercises within particular sports, and other variables related to performance except for body balance (e.g., core strength and stability, muscle power and strength, etc.). Altogether 193 papers were found through database searching and other sources when these keywords were connected with particular sports. After an initial screening and assessing for eligibility, studies that did not meet the inclusion criteria were removed. Out of 126 articles included in this scoping review, only 14 investigated the association between variables of postural balance control and measures of sport-specific performance. Figure 1 shows phases of the search process.

## 3. Results and discussion

In summary, studies in following sports were reviewed:

(a) shooting and air-rifle shooting (Niinimaa and McAvoy, 1983; Larue et al., 1989; Aalto et al., 1990; Era et al., 1996; Ball et al., 2003; Mononen et al., 2007; Ihalainen et al., 2016a,b; Spancken et al., 2021; Lang and Zhou, 2022a,b), air-pistol shooting (Ko et al., 2017, 2018), sharpshooting (Konttinen et al., 1998, 1999), small-bore shooting (Spancken et al., 2021);
(b) biathlon (Niinimaa and McAvoy, 1983; Larue et al., 1989; Ihalainen et al., 2018; Michalska et al., 2022);
(c) archery (Mason and Pelgrim, 1986; Stuart and Atha, 1990; Mohamed and Azhar, 2012; Spratford and Campbell, 2017; Musa et al., 2018; Serrien et al., 2018; Simsek et al., 2019; Wada and Takeda, 2020; Sarro et al., 2021; Vendrame et al., 2022);
(d) gymnastics (Bringoux et al., 2000; Vuillerme et al., 2001a,b; Aydin et al., 2002; Asseman et al., 2004, 2005, 2008; Davlin, 2004; Bressel et al., 2007; Carrick et al., 2007; Croix et al., 2010; Omorczyk et al., 2018; Marcolin et al., 2019; Busquets et al., 2021), rhythmic gymnastics (Kioumourtzoglou et al., 1997; Calavalle et al., 2008), acrobatic gymnastics (Sobera et al., 2019; Gómez-Landero et al., 2021; Opala-Berdzik et al., 2021), and artistic gymnastics (Puszczałowska-Lizis and Omorczyk, 2019);
(e) dancing (Perrin et al., 2002; Gerbino et al., 2007; Stins et al., 2009; Munzert et al., 2019; Nikolaidou et al., 2021), and ballet dancing (Schmit et al., 2005; Michalska et al., 2018; Thalassinos et al., 2018);
(f) golf (Lephart et al., 2007; Sell et al., 2007; Wells et al., 2009; Glofcheskie and Brown, 2017);
(g) baseball (Marsh et al., 2004; Butler et al., 2016; Liang et al., 2019);
(h) basketball (Perrin et al., 1991; Kioumourtzoglou et al., 1998; Bressel et al., 2007; Matsuda et al., 2008; Verhoeven and Newell, 2016; Halabchi et al., 2020; Glass and Ross, 2021; Makaracı et al., 2021);
(i) handball (Caballero et al., 2020);
(j) ice hockey (Behm et al., 2005; Kim et al., 2018; Rosker et al., 2021);
(k) soccer (Davlin, 2004; Paillard and Noé, 2006; Paillard et al., 2006; Bressel et al., 2007; Gerbino et al., 2007; Matsuda et al., 2008; Thorpe and Ebersole, 2008; Ben Moussa et al., 2012; Pau et al., 2015, 2018; Edis et al., 2016, 2017; Ozmen, 2016; Thalassinos et al., 2018; Jadczak et al., 2019a,b; Liang et al., 2019; Halabchi et al., 2020; Snyder and Cinelli, 2020; Zago et al., 2020; Glass and Ross, 2021; Śliwowski et al., 2021; González-Fernández et al., 2022; Scinicarelli et al., 2022);
(l) tennis (Caballero et al., 2021; Glass and Ross, 2021; Kozinc and Šarabon, 2021);
(m) volleyball (Agostini et al., 2013; Borzucka et al., 2020a,b; Makaracı et al., 2021);
(n) judo (Paillard et al., 2002, 2007b; Perrin et al., 2002);
(o) alpine skiing (Noé and Paillard, 2005; Kiers et al., 2022), cross country skiing (Glass and Ross, 2021), and snowboarding (Platzer et al., 2009a,b);
(p) surfing (Chapman et al., 2008; Paillard et al., 2011), canoeing and kayaking (Stambolieva et al., 2012), and paddle boarding (Schram et al., 2016);
(r) swimming (Davlin, 2004; Matsuda et al., 2008);
(s) others, such as track and field (Schmit et al., 2005), cascade ball juggling (Rodrigues et al., 2016), long distance running (Glofcheskie and Brown, 2017), running (Leightley et al., 2017), horseback riding (Olivier et al., 2019), pentathlon (Sadowska et al., 2019), and slacklining (Kodama et al., 2021).

### 3.1. Sport-specific training induced adaptations in postural balance control and performance measures

A review of the literature revealed that training under sport-specific conditions that include various balance exercises can lead to an improvement of postural stability. A recent analysis of postural sway variables in 936 athletes ranging from 6 to 47 years (shooters, football players, boxers, cross-country skiers, gymnasts, runners, team sport players, wrestlers, tennis players, alpine skiers, rowers, speed skaters and figure skaters) identified that practicing any kind of sport improves bipedal balance (Andreeva et al., 2021). However, it mostly depends on their age, and partly on their level of performance, sex, and shoe features (Andreeva et al., 2020). Usually, balance performance is associated with the level of competition, with better balance in more proficient athletes (Hrysomallis, 2011).

Most studies compared postural sway variables in athletes of different sports and levels of expertise, or physically active individuals with a control group of sedentary individuals. In general, athletes are superior to non-athletes in balance performance (Davlin, 2004). More specifically, athletes of both team and individual sports demonstrate better body balance over the group of non-athletes (Mocanu et al., 2022). Lateral CoP deviation is also lower in individuals with moderate than low physical activities (Onofrei and Amaricai, 2022). However, static balance in collegiate athletes with a sport background does not differ from their multisport counterparts (Chou et al., 2022).

Further studies have been conducted to investigate acute (Zemková and Hamar, 2014; Zemková, 2022) and adaptive changes in postural balance control. A recent scoping review revealed that neuromuscular control of core and postural stability contributes to more effective functional movements in a given sport (Zemková and Zapletalová, 2022). Including sport-specific and general core and balance exercises into training programs improves postural stability, strength and endurance of the back muscles (Zemková and Zapletalová, 2022). Although the ankle is the most significant predictor of the magnitude of body sway, the trunk is the second most important element during specific postural tasks (Duchene et al., 2021). Postural control of the trunk expressed by lower CoP movement in a seated position is better in long distance runners and golfers than in controls (Glofcheskie and Brown, 2017). Lumbar spine angular displacement is lower, muscle activation amplitudes is higher, and trunk muscle activation onset is faster in response to unexpected perturbations of the trunk in athletes than in controls (Glofcheskie and Brown, 2017). Variable and absolute error in the trunk repositioning task is lower in golfers than in runners and controls, indicating higher proprioceptive ability (Glofcheskie and Brown, 2017). This assumes that an association exits between proprioception, postural and neuromuscular control in athletes, and that it discriminates between those with different training background (Glofcheskie and Brown, 2017).

Balance training can improve some sport-related performance measures, however its transfer to general functional enhancement is unclear (Yaggie and Campbell, 2006). Balance adaptations are specific to practiced physical activities (Paillard, 2017). Successful competitive athletes have the best postural stability in both specific and decontextualized balance conditions in relation to sports practiced (Paillard, 2019). There is only small transfer to a non-experienced task (Paillard, 2017). The level of performance is often associated with proprioceptive acuity in elite athletes (Han et al., 2015). However, the relationship between sport-specific training and improvements in proprioceptive acuity is limited by biologically determined factors (Han et al., 2015). An investigation of the effects of sport-specific training on CoP velocity and displacement in collegiate cross country, basketball, and soccer athletes indicates that between-group differences may be related to sensorimotor adaptations in a given sport, including proprioceptive acuity, strength and power of lower limbs, and efficiency of vestibular system (Glass and Ross, 2021). The involvement of muscle synergies or cutaneous feedback are further potential mechanisms (Glass and Ross, 2021). For instance, the use of vestibular and proprioceptive cues in contact sports athletes leads to better postural stability (Liang et al., 2019). Furthermore, postural strategies are better in athletes at higher than lower competition level (Paillard, 2019). Dynamic balance strategies may be influenced by neurocognitive performance in athletes (Porter et al., 2022). Different approaches may be used when performing difficult balance tasks with strategies related to higher anteroposterior and lower vertical acceleration in higher than lower neurocognitive performers (Porter et al., 2022).

In comparison with a number of studies dealing with neurophysiological adaptations in postural control induced by general body conditioning exercises, scarce research has been conducted to investigate adaptive changes in postural balance control in response to sport-specific balance exercises and their effects on athletic performance.

#### 3.1.1. Shooting

Static balance is important for performance in shooters. Both postural sway and rifle stability are different in national- and elite-level air-rifle and small-bore shooters (Spancken et al., 2021). While aiming accuracy, rifle stability, and aiming time influence the shot score in national-level air-rifle athletes, postural sway does not affect the shot score in these athletes (Spancken et al., 2021). Sway velocity in shooters is reduced under both visual and non-visual conditions when using competition clothing (Aalto et al., 1990). Shooters use a higher amount of proprioceptive and vestibular cues for stabilizing their posture (Aalto et al., 1990). This posture stabilization is better in top-level male shooters compared to national-level male and top-level female shooters, whose stability is better than in naive shooters (Era et al., 1996). In particular, the ability to stabilize posture prior to the shot is better in experienced shooters (Era et al., 1996). However, worse stabilization of posture does not contribute to bad results among the highly trained shooters (Era et al., 1996).

In addition to the CoP movement, also arm joints and the pistol end point motions are lower in expert than in novice shooters (Ko et al., 2017, 2018). Kinematic variables are reduced in a lower dimensional functional unit by skill acquisition, so pistol-aiming is characterized by upper limb and posture performance (Ko et al., 2017). The coordination of pistol and posture motion is more variable in novice shooters, whereas it is more consistent in the skilled group (Ko et al., 2018). Dispersion and complexity is reduced in the skilled arm-pistol motion (Ko et al., 2018). Thus, rifle motion is an important factor of advanced performance in shooting (Konttinen et al., 1998). While pre-elite athletes rely more on the visual-spatial processing, elite athletes focus more on stabilization of rifle position by using psychomotor regulation (Konttinen et al., 1998). There is a relationship of postural sway velocity and amplitude with changes in the concomitant brain slow potential, however it depends on the expertise of shooters (Konttinen et al., 1999). Reduced postural sway amplitude coincides with decreased frontal positivity in elite shooters, whereas postural anteroposterior sway velocity and amplitude are characterized by the central negativity lateralization in the non-elite shooters (Konttinen et al., 1999).

#### 3.1.2. Biathlon

The strategy of biathletes is different from the one used by rifle shooters due to adaptive changes of their respective specializations (Larue et al., 1989). Postural stability is important during standing and aiming an air rifle at both rest and during a cross-country ski racing. Anteroposterior sway movement during standing at rest is about twice that of movement in a mediolateral direction (Niinimaa and McAvoy, 1983). However, lateral sway movement increases with aiming, and both sway movements are similar after exercise (Niinimaa and McAvoy, 1983). Vertical hold stability and cleanness of triggering are the most important factors influencing shooting performance at rest as well as during competition shots (Ihalainen et al., 2018). Postural stability in shooting direction is associated with vertical hold stability and cleanness of triggering (Ihalainen et al., 2018). Cleanness of triggering, aiming accuracy, vertical and horizontal hold stability decrease from resting to competition simulation shots, accompanied by a decrease in postural balance (Ihalainen et al., 2018). Therefore, biathletes should focus on vertical hold stability and cleanness of triggering to improve their shooting performance (Ihalainen et al., 2018). Better postural stability in shooting direction can contribute to the improvement of these technical components (Ihalainen et al., 2018). National- and junior-team biathletes differ only in the percentage of hits during resting shots and postural stability on the left leg in shooting direction during competition shots, but intensive exercise affects these technical components in both groups of shooters (Ihalainen et al., 2018). Different motor control strategy is used in experienced biathletes, beginners and controls, characterized by better postural stability during aiming at the target and shooting (Michalska et al., 2022). Body sway is significantly smaller in the position shooters than in those with less than four months of training (Niinimaa and McAvoy, 1983).

#### 3.1.3. Archery

Maximum sway speed, draw force, and clicker reaction time are important factors of shot performance in elite recurve archers (Spratford and Campbell, 2017). More specifically, reducing postural sway during the release phase increases shooting performance of skilled archery athletes (Mohamed and Azhar, 2012). In addition to reduced postural sway speed, also greater bow draw force and reduced clicker reaction time post-arrow release contribute to better scoring shots (Spratford and Campbell, 2017). The ability to control the bow and postural sway movements together with higher activation of muscle extensor digitorum is better in medalist and elite archers (Musa et al., 2018). Postural sway during arrow shooting is lower in elite archers compared to those of mid-level and beginners (Simsek et al., 2019). Expert archers tend to maximize postural stability and develop personal strategies of muscular activation and time management (Vendrame et al., 2022). There is a considerable variability in the precision with which the positions of head, elbow and bow at the moment of loose are replicated by archers of similar skills level (Stuart and Atha, 1990). It seems that precise postural consistency is not the primary feature distinguishing between the performance of archers at the higher skill levels (Stuart and Atha, 1990). Serrien et al. (2018) suggets that it is not necessary for elite archers to minimize the movements of all degrees of freedom during aiming, but rather that the structure of variability of the redundant kinematic chain is exploited so that the relevant performance variable (orientation of the arrow) is stabilized. Taking into account better stability against visual disturbance in archers than ball game players and untrained subjects, one may assume that they rely on proprioceptive inputs to maintain balance (Wada and Takeda, 2020). Their training re-weights sensorimotor dominance from vision to proprioception for posture regulation to increase shooting accuracy (Wada and Takeda, 2020).

#### 3.1.4. Gymnastics

Expertise in gymnastics enhances postural stability only in situations where it is practised, i.e., while standing on one leg with eyes open (Asseman et al., 2008). The CoP excursion during one-legged stance is lower in base than top gymnasts, whereas their values are lower in mid- than early adolescent gymnasts, regardless of the task (Gómez-Landero et al., 2021). However, the level of expertise does not have effect on bipedal postural stability (Marcolin et al., 2019). Therefore, sport-specific tasks are more selective in representing the expertise level in gymnasts (Marcolin et al., 2019).

Gymnasts have greater postural stability than non-gymnasts (Carrick et al., 2007). Specifically, decreasing CoP displacements by reinsertion of proprioceptive information is better in gymnasts than non-gymnasts (Vuillerme et al., 2001b). Greater gymnastic skills are characterized by more stable, less regular, and less variable acceleration time-series (Lamoth et al., 2009). Gymnastic training improves the ability to change the frame of reference (Croix et al., 2010). The rod-and-frame test results correlate with postural stability, and experts are less field dependent than non-experts (Croix et al., 2010). The remaining sensory modalities are used more efficiently under eyes closed conditions in expert gymnasts (Croix et al., 2010). They use remaining sensory modalities mainly when standing on unstable surfaces with eyes closed (Vuillerme et al., 2001a). Specific training in gymnasts improves the efficiency of the integration process leading to the perception of the body orientation in space (Bringoux et al., 2000). The relevance of otolithic and/or interoceptive inputs increases with their expertise (Bringoux et al., 2000).

Age influences static balance in acrobatic gymnastics (Gómez-Landero et al., 2021). Proprioceptive reweighting processes can be improved by gymnastic training during childhood, leading to similar control and coordination of posture as adults (Busquets et al., 2021). More specifically, better anteroposterior postural stability with eyes open is related to greater age, body mass and height, biological maturity, and training experience in artistic gymnasts, whereas better postural stability regardless of visual conditions is related to higher body mass and BMI percentiles in acrobatic gymnasts (Opala-Berdzik et al., 2021).

Static and dynamic balance, and whole-body coordination are better in elite rhythmic gymnasts than in controls (Kioumourtzoglou et al., 1997). Static balance, hand-eye coordination, and an anticipation of coincidence (hand-eye coordination and its anticipation on visual accuracy) are better in older than younger (13–15, 11–12, 9–10 years) elite rhythmic gymnasts (Kioumourtzoglou et al., 1997). Moreover, strategies in lateral directions during simple postural tasks are better in rhythmic gymnasts than in students, indicating that their training has a direct impact on mediolateral bipedal balance (Calavalle et al., 2008). Their training also improves postural stability, sense of ankle joint position, and increases muscle tone (Aydin et al., 2002). Gymnasts learn and perform better in new motor routines compared to those with high fatigability ratios and lower adaptability scores (Carrick et al., 2007).

The stability of standing and handstand is differentiated by the sport advancement level so that the control of both body positions is better in seniors than in juniors (Omorczyk et al., 2018). In particular, more experienced gymnasts have better postural performance during the handstand than non-experts (Croix et al., 2010). Exerting force on a floor surface helps to minimize body sway, however less experienced athletes are not able to do that even after long-term training (Sobera et al., 2019). More skilled performance is demonstrated by adaptation of reactive rather than anticipatory strategies. Mechanical advantages in mediolateral balance while standing on wider base of support are observed in more skilled athletes than in less (Wyatt et al., 2021). Controlling the mediolateral CoP movement with eyes open is not essential for body stability in the frontal plane in seniors practicing gymnastics (Puszczałowska-Lizis and Omorczyk, 2019). The ratios of CoP velocity with eyes closed on eyes open are similar in the bipedal and handstand postures, suggesting that the specificity or difficulty of the posture is not directly related to the effect of vision removal (Asseman et al., 2005). However, stability indices in standing are not related to those of the handstand, indicating that stability in a standing position does not predict handstand performance (Omorczyk et al., 2018). On the contrary, handstand stability is not transferable to upright standing postures among elite gymnasts (Asseman et al., 2004).

#### 3.1.5. Dancing

Postural sway is more stationary (lower absolute trend), less stable (lower maxline), less regular (lower recurrence), and less complex (lower entropy) in ballet dancers than in track athletes (Schmit et al., 2005). Dancers’ stability is also better than in soccer players (Gerbino et al., 2007). Both professional dancers and high-level judoists perform better than controls, which indicates that their training has positive effects on sensorimotor adaptabilities (Perrin et al., 2002). Higher trembling component during both quiet and inclined standing indicates better ability to maintain balance under unstable conditions in professional ballet dancers than in non-trainees (Michalska et al., 2018). An expert advantage on postural stability is observed in specific dance tasks but not in static everyday tasks (Munzert et al., 2019).

However, postural stability may also play a role in performance of less trained individuals, such as entry level university dancers (Misegades et al., 2020) or older dancers (Nikolaidou et al., 2021). The superior balance performance in dancers over 70 years old most likely results from coordinated intersegmental movements, including between-leg alternations during dancing (Nikolaidou et al., 2021).

#### 3.1.6. Baseball, basketball, handball, hockey, tennis, volleyball

Sport-specific balance in team sports athletes may depend on their expertise and level of performance. For example, dynamic balance depends on the competition level of baseball players (Butler et al., 2016).

Dynamic balance is lower and hand coordination is higher in elite male basketball players than in controls. They are also better on prediction measures, selective attention, and memory-retention (Kioumourtzoglou et al., 1998). The coordination of ball release and postural stability is important for success in shooting changes, reflecting the level of athletic skills (Verhoeven and Newell, 2016). Interestingly, the best basketball player has significant results in static balance with no differences in eyes open and eyes closed conditions (Perrin et al., 1991).

Better balance in experienced handball players is associated with the maturation of the motor system rather than performance level (Caballero et al., 2020). Slower CoP velocity during balance tasks and less irregular movements are in players who throw with less accuracy, whereas less auto-correlated and more irregular CoP movements are in players who throw faster (Caballero et al., 2020). Less dependence on previous behavior (lower regularity and long-range auto-correlation, respectively) has been considered as higher flexibility to perform motion adjustments to reduce motor output error (Wang and Yang, 2012). This means that expert players display a more exploratory behavior when performing balance tasks, thus have a greater adaptive capacity of the CNS over longer time scales (Barbado Murillo et al., 2017). Similarly, high postural strategies used for readjustments of unexpected perturbations are found in elite ice hockey players (Kim et al., 2018). Their muscle synergies display low co-activation strategy of antagonists and agonists in the neck and ankle (Kim et al., 2018). Upper body loading and sport-specific posture in elite ice hockey players induce adaptations in neck proprioception (Rosker et al., 2021). Cervical spine afferent input is esential for maintaining unilateral balance in hockey players (Rosker et al., 2021). Therefore, neck kinaesthesia, in addition to postural control, may influence oculomotor performance, which is important for initiating eye movement changes (Rosker et al., 2021).

Static balance is only slightly different on the non-preferred and preferred leg determined by the manipulation task in the form of kicking a ball in highly-trained tennis players, whereas there are no differences when the classification is based on the preference for performing a single-leg jump (Kozinc and Šarabon, 2021). Similarly, there are no between-leg differences in dynamic balance for the landing task (Kozinc and Šarabon, 2021).

The model of sensory integration in postural balance control is different in volleyball players and non-athlete controls (Agostini et al., 2013). Between-group postural stability is different with eyes open but not with eyes closed, which may be ascribed to better dynamic visual acuity in athletes because static refractive errors are corrected in both groups (Agostini et al., 2013).

Parallel stance and dominant leg postural sway is better in deaf volleyball than basketball players, whereas there are no significant between-group differences in non-dominant leg postural sway (Makaracı et al., 2021). Postural regulation in top level male volleyball players is more precise and less vulnerable to external disturbances than in non-athletes, which support optimal timing and precision of actions (Borzucka et al., 2020a). They have better capacity to use postural strategies for maintenance of balance and reduce the use of proprioception for performing challenging postural and motor tasks (Borzucka et al., 2020a). Volleyball players develop a unique posture control resulting from the motor demands of this sport (Borzucka et al., 2020b).

#### 3.1.7. Soccer

Dynamic balance is better in gymnasts than in soccer players and swimmers, whereas there are no differences between two remaining groups of athletes (Davlin, 2004). However, Bressel et al. (2007) found that dynamic balance is not different in soccer players and gymnasts. Differences in these findings may be mainly ascribed to different tests used. While in the first case, a stabilometer, which requires participants to continuously adjust posture to maintain an unstable platform in the horizontal position, was used (Davlin, 2004), in the second case, participants performed multidirectional maximal single-leg reaches from a unilateral base of support using the Star Excursion Balance Test (Bressel et al., 2007). Furthermore, soccer players have superior postural control compared to baseball players and untrained students (Liang et al., 2019). Their ability to maintain one-legged balance is also better than in swimmers, basketball players, and non-athletes (Matsuda et al., 2008). Basketball players present inferior dynamic balance in comparison with soccer players and inferior static balance in comparison with gymnasts (Bressel et al., 2007). However, dancers perform better than soccer players (Gerbino et al., 2007).

Postural parameters improve with age until zero maturity offset is achieved (Zago et al., 2020). It seems that these parameters are most stable in developing soccer players (Zago et al., 2020). Variance in multidirectional speed performance in young soccer players may be predicted from dynamic balance performance and chronological age (Scinicarelli et al., 2022). There is a strong association of multidirectional speed performance with dynamic balance performance of the dominant side, whereas there is a small relationship with limb symmetry index (Scinicarelli et al., 2022).

Postural strategy and performance is influenced by the competition level in soccer players. Playing experience also affects postural measures and strategies in test conditions specific to playing soccer (Paillard et al., 2006). Moreover, static balance varies in elite soccer players playing at different positions, i.e., it is better in midfield players than those in other positions (Jadczak et al., 2019b), whereas centre-backs are worse than wingers and forwards (González-Fernández et al., 2022). Postural stability, postural strategy, and the use proprioception and vision information is different in national and regional soccer players (Paillard et al., 2006). Postural stability is less disturbed when manipulating sensory information in the high-level than in the regional-level players (Paillard et al., 2007a). The internal model of verticality is better in the high-level than in the regional-level players. Those with better postural stability are less disturbed by sensorial manipulation than the others (Paillard et al., 2007a). Proprioceptive executive control is improved by soccer-specific training, resulting in better correlation between CoP and single-support balance during a dynamic visuomotor reaching task of lower limbs (Snyder and Cinelli, 2020). Visual contribution is lower in professional than amateur players (Ben Moussa et al., 2012). Professional soccer players are less dependent on vision when controlling their posture, so vision can be dedicated to treat with information during the game (Paillard and Noé, 2006).

Professional soccer players have also greater postural stability on the non-dominant leg (Jadczak et al., 2019b). Their balance control is widely influenced by concentric isokinetic strength (peak torque of quadriceps and peak torque of hamstrings at high angular velocity), particularly in the supporting, non-dominant leg (Śliwowski et al., 2021). Better balance in young national-level soccer players is characterized by more efficient and faster stabilization after a forward jump, whereas the unipedal stance test is not able to reveal differences in postural control associated with a combination of physical and technical skills (Pau et al., 2018). Postural balance control is among the most important factors that influence performance of technical skills under the pressure and unexpected changing situations in trained amateur soccer players (Edis et al., 2016). Therefore, a combined training involving soccer-specific and balance exercises can significantly contribute to their performance (Edis et al., 2017). The higher their sport level, the better their balance. This may indirectly contribute to successful performance in any game situations (Jadczak et al., 2019a).

In practice, both static and dynamic balance tests should be performed in soccer players because balance variables in these two conditions are not related (Pau et al., 2015). For instance, Balance Error Scoring System including static postures reflects deficits in postural control better than dynamic balance tests in professional football and basketball players (Halabchi et al., 2020). The measures from the Star Excursion Balance Test may not reflect the balance performance in well-trained football and basketball players who have a better balance when performing sport-related skills (Halabchi et al., 2020). However, soccer players reach significantly farther than the non-soccer athletes, suggesting that the Star Excursion Balance Test may be sensitive to training status and/or sport-related adaptations (Thorpe and Ebersole, 2008). This a unilateral, functional joint-stability task may reflect their adaptation to single-leg exercises and other sport-related skills, such as standing on one leg while kicking the ball. Values of this test are not associated with those of the side bridge, trunk extension and flexion tests, which indicates that core stability does not contribute significantly to dynamic balance (Ozmen, 2016).

#### 3.1.8. Golf

Balance, core strength and stability, peripheral muscle strength, and flexibility correlate with performance in golf (Wells et al., 2009). These abilities including balance, flexibility and strength are improved through a specific exercise program in golf, which results in higher upper-torso axial rotational velocity, and consequently also in higher club head and ball velocity, as well as driving distance (Lephart et al., 2007). Balance, torso, shoulder and hip strength and flexibility are better in golfers with handicap (HCP) of < 0 than in those with HCP of 10–20 (Sell et al., 2007).

#### 3.1.9. Combat sports (judo)

Practice of high-skill activities that include proprioceptive afferences enhances both postural stability and performance (Perrin et al., 2002). Dancers and judoists perform better than controls because their training improves sensorimotor functions (Perrin et al., 2002). Specific postural adaptations are also induced by different movements performed on one or two legs (a tokui-waza in monopodal and bipodal stance) in competition-level judoists (Paillard et al., 2007b). However, static balance does not differ significantly between regional and national and international level judoists (Paillard et al., 2002).

#### 3.1.10. Water sports (canoeing, kayaking, paddle boarding, surfing)

The model of sensory integration is different in young kayakers and canoeists than in non-athletes as a result of their sport specializations, which may be attributed to re-adaptation deficit after disembarking to stable surface with diminished sensitivity of the vision and vestibular systems (Stambolieva et al., 2012).

Stand-up paddle boarding athletes have increased static and dynamic balance, aerobic and anaerobic fitness, and isometric trunk endurance (Schram et al., 2016).

There is an association of postural stability with the competition level of surfers (Paillard et al., 2011). The sensorimotor dominance for maintenance of balance can be shifted from vision to proprioception in expert surfers (Paillard et al., 2011). However, standard postural sway variables are not able to indicate whether surfing expertise facilitates balance adaptations (Chapman et al., 2008).

#### 3.1.11. Winter sports (alpine skiing)

Relative dynamic postural stability index improves annually in competitive youth skiers (Kiers et al., 2022). However, age and biological maturation correlate with absolute but not with relative values of dynamic postural stability index (Kiers et al., 2022). Furthermore, postural stability is similar when tested in ski boots and it is similarly influenced by the absence of visual information in regional and national level skiers (Noé and Paillard, 2005). However, postural stability without ski boots is better in regional than national level skiers (Noé and Paillard, 2005). Such an inferior postural stability may be attributed to repetitive wearing of ski boots during a long-term training, which affects balance by restricting the ROM of the ankle-foot complex (Noé and Paillard, 2005).

#### 3.1.12. Other sports

Practicing many other sports may contribute to the improvement of balance, however a significant relationship with athletic performance has been rarely documented. For instance, proprioceptive functions of posture and postural muscle tone during bipodal dynamic perturbations are developed by horseback riding (Olivier et al., 2019). Refined postural stability is also associated with expertise in cascade juggling (Rodrigues et al., 2016). Stance stability is better in pentathletes than in untrained individuals and they are also less dependent on vision (Sadowska et al., 2019). Experts in slacklining tend have a more antiphase coordination pattern and coordinate their hands more sustainably than novices (Kodama et al., 2021). Postural control declines in master runners, however they may benefit from balance exercises (Leightley et al., 2017).

### 3.2. The relationship between postural balance control and athletic performance

Analysis of the literature revealed that postural balance control is a key determinant of performance in several sports (Zemková, 2014b). However, postural sway variables have been found to be associated with only a few measures of sport-specific performance (Table 1).

**TABLE 1:** An overview of studies dealing with the relationship between postural sway variables and measures of athletic performance.

Authors (year)	Study objective	Participants	Postural sway variables	Athletic performance measures	The relationship between postural sway variables and measures of athletic performance
Mason and Pelgrim (1986)	To quantify body movements of the archers and to identify their relationships with shooting accuracy	Austrian senior and junior archers	CoP movement in Y and X directions before and after arrow release	Arrow shooting accuracy	A significant correlation (-0.30) between shooting performance and the total excursion of the archer’s CoP in the interval one second prior to arrow release; The total CoP excursion correlates with the criterion variable of shooting performance in junior (-0.51) but not in senior archers (-0.24)
Ball et al. (2003)	To examine the relationships between body sway, aim point fluctuation and performance in rifle shooting on an inter- and intra-individual basis	Six elite shooters	Body sway parameters quantified for the time periods 5 s to shot, 3 s to shot and 1 s to shot	Four aim point fluctuation parameters quantified for the time periods 5 s to shot, 3 s to shot and 1 s to shot	Body sway is related to aim point fluctuation; These relationships are specific to the individual, with the strength of association, parameters of importance and time period of importance different for different shooters
Marsh et al. (2004)	To examine the relationship between balance and pitching error in college baseball pitchers	Sixteen college baseball pitchers, 9 National Association of Intercollegiate Athletics and 7 National Collegiate Athletic Association, Division III	Average sway velocity during dominant leg unilateral stance with eyes open and eyes closed using the Balance Master System 7.04; Sensory organization testing on the SMART EquiTest System providing information on the use of the somatosensory, visual, and vestibular inputs	Pitching error assessed with a high-speed video camera recorder; Pitch velocity measured using a JUGS radar gun	A significant negative correlation between sensory organization test 5 and pitching error (-0.50) and between sensory organization test 5/1 and pitching error (-0.50); A positive correlation between unilateral stance eyes closed and a pitch velocity (0.52); No significant correlation between unilateral stance eyes open and pitching error (-0.24) or unilateral stance eyes closed and pitching error (-0.29)
Behm et al. (2005)	To determine the relationship between specific performance measures and hockey skating speed	Thirty competitive secondary school and junior hockey players	Balance ratio (wobble board test)	Maximum skating speed (a 40-yd (36.9-m) sprint)	Significant correlations between skating performance and the sprint and balance tests; Significant correlations between balance and players under the age of 19 years (-0.65) but not those over 19 years old (-0.28)
Mononen et al. (2007)	To examine the relationships between shooting accuracy and shooters’ behavioral performance, i.e., postural balance and gun barrel stability, among novice rifle shooters in intra- and inter-individual levels	Fifty-eight shooters	Postural balance assessed in terms of anteroposterior [VEL(AP)]and mediolateral [VEL(ML)] sway velocity of the CoP movement	Rifle stability assessed in terms of horizontal [DEV(H)] and vertical [DEV(V)] deviation of the aiming point	The shooting accuracy is related to postural balance and rifle stability, but only at the inter-individual level; The correlation coefficients between shooting score and behavioral performance variables range from -0.29 to -0.45; The VEL(ML) and the DEV(H) as independent variables account for 26% of the variance in the shooting score; Postural balance is related to the shooting accuracy both directly and indirectly through rifle stability
Platzer et al. (2009a)	To assemble and evaluate a battery of tests for the snowboard disciplines parallel, snowboard cross (SBX), big air, and half-pipe (HP)	Thirty-seven competitive snowboarders	Dynamic unipedal balance measured by the Biodex Balance System	World Cup & International Federation of Skiing points	Dynamic unipedal balance is not associated with snowboarders’ ranking points
Platzer et al. (2009b)	To evaluate the influence of different physiological factors on the luge start and identify an appropriate physiological test battery	Thirteen male members of the Austrian national luge team	Dynamic unipedal balance measured by the Biodex Balance System	Starting speed measured by the luge start simulator	Dynamic unipedal balance is associated with end speed (0.590) but not with maximal speed
Wells et al. (2009)	To identify physiological correlates of golf performance in elite golfers under laboratory and tournament conditions	Elite golfers	Timed unipedal stance	Ball speed and distance, average score, greens in regulation, short game measures, and putting accuracy	Static balance is associated with greens in regulation (-0.43) and average putt distance after a chip shot (0.50)
Ihalainen et al. (2016a)	To identify the most important factors determining performance in elite-level air rifle shooting technique	Elite-level air rifle shooters		Six components in the air rifle shooting technique: aiming time, stability of hold, measurement time, cleanness of triggering, aiming accuracy, and timing of triggering	Stability of hold, cleanness of triggering, aiming accuracy, and timing of triggering are the most important predictors of shooting performance, accounting for 81% of the variance in shooting score; The direct effect of postural balance on performance is small, accounting for less than 1% of the variance in shooting score; The effect can be greater through a more stable holding ability, to which postural balance is significantly correlated
Ihalainen et al. (2016b)	To describe the long-term changes in shooting technique in relation to competition performances in elite air-rifle shooters	Seventeen elite air rifle shooters	Postural-balance variables measured with force platform	Shooting score and aiming-point-trajectory variables obtained with an optoelectronic shooting device; Shooters’ competition results collected from all international and national competitions during the 3-y period	Seasonal mean test results in stability of hold (-0.70) and cleanness of triggering (-0.75) are related to competition performances; Changes in stability of hold (-0.61) and cleanness of triggering (-0.39) are related to the changes in competition performances; Postural balance in shooting direction is more related to cleanness of triggering (0.57), whereas balance in cross-shooting direction is more related to stability of hold (0.70)
Caballero et al. (2021)	To assess the relationship between balance and tennis performance using linear and non-linear parameters through 1) the comparison of tennis players of different ages and levels of expertise, and 2) analyzing the relationship between balance and tennis serving speed and accuracy	One hundred and six recreational and expert male tennis players	Temporal dynamics of postural control during a balance task on an unstable surface analyzed through the mean velocity and the detrended fluctuation analysis (DFAV) of the CoP	Tennis serve performance quantified by measuring accuracy and speed	The CoP showed a reduction of auto-correlated variability (reflected by DFAV) with age but mainly in expert players; The CoP dynamics is the only balance parameter discriminating sport expertise and it is related to age; Sport experience in expert tennis players induces balance adaptations characterized by a higher ability to perform postural adjustments; The lack of correlations suggests that balance, measured with scattering variables, in a non-specific task is not a main determinant of sport performance in tennis serve
Lang and Zhou (2022a)	To examine the relationships between postural balance, aiming technique and shooting score among elite rifle shooters at an intra- and inter-individual level	Twelve elite athletes belonging to China national team	Postural balance variables measured using footscan 1.0 force platform	Aiming technique parameters measured using a SCATT MX-02 optoelectronic training device	Inter-individual analyses: Postural balance is negatively correlated with shooting score (-0.697) and aiming accuracy (-0.810); A positive correlation of postural balance with the stability of hold (0.923) and stability of triggering (0.564); Intra-individual analyses: A significant correlation between postural balance and performance, aiming accuracy and stability of hold; Postural balance is related to the stability of triggering; Postural balance is not significant with aiming time on an intra- and inter-individual basis
Sarro et al. (2021)	To investigate the relationship between bow stability and postural control in recurve archery according to shooting performance	Eight archers	The CoP position of the archer (the point of application of the resultant ground reaction force on a force plate) measured during the aiming phase, representing archer displacement	The three-dimensional position of one marker attached to the bow measured during the aiming phase, representing bow displacement	A significant correlation between CoP and bow displacement in the direction toward/away from the target (COP_X_ and D_X_) and between COP_X_ and vertical displacement of the bow (D_Z_) during the highest scoring shot
Lang and Zhou (2022b)	To identify the determinants of shooting performance in elite 10 m air rifle shooters	Twelve international-level 10 m air rifle shooters belonging to China’s national team	A footscan 1.0 force platform used to collect postural balance parameters (A-P and M-L balance)	A SCATT MX-02 optoelectronic shooting test system used to collect shooting score and shooting technical variables (holding and aiming, stability of triggering, time)	The holding and aiming ability is the most important component, which could explain the 36.3% variance of shooting performance; The stability of triggering is the second important component, which could explain the 24.5% variance of shooting performance

Static bipedal balance is associated with shooting accuracy in rifle shooters (Ball et al., 2003) but only at the inter-individual level (Mononen et al., 2007). While inter-individual analyses revealed that postural stability negatively correlates with aiming accuracy and shooting score and positively correlates with triggering and hold stability, intra-individual analyses showed the relationship between postural stability and performance, hold and triggering stability, and aiming accuracy (Lang and Zhou, 2022a). Thus, aiming and holding are the essential factors of shooting performance, followed by the stability of triggering (Lang and Zhou, 2022b). The aiming accuracy, stability of hold, cleanness and timing of triggering are key predictors of shooting performance, whereas postural stability plays a very small role (Ihalainen et al., 2016a). The effect is higher through stable holding, which correlates with postural stability (Ihalainen et al., 2016a). Cleanness of triggering and stability of hold are also associated with competition performances in elite air-rifle shooters (Ihalainen et al., 2016b). The stability of hold is also associated with postural balance control in cross-shooting direction, whilst in shooting direction it is related to cleanness of triggering (Ihalainen et al., 2016b). A relationship between body sway and aim point fluctuation means that aim point fluctuation increases and performance decreases when body sway increases (Ball et al., 2003). Specifically, the CoP velocity in mediolateral direction and the aiming point deviation are independent variables explaining the shooting score (Mononen et al., 2007).

Similarly, postural sway displacement in the X direction prior to the arrow release is associated with performance score in archers (Mason and Pelgrim, 1986). There is a significant but low inverse relationship between the total excursion of the archer’s CoP in the interval one second prior to arrow release and shooting performance (Mason and Pelgrim, 1986). This association of the total CoP excursion with the criterion variable of shooting performance is stronger in junior than senior archers (Mason and Pelgrim, 1986). That is, shooting performance in less experienced archers is better when their postural sway movement decreases (Mason and Pelgrim, 1986). Therefore, the synchronization of body and bow sway is important for shot accuracy in recurve archers, which may be corroborated by a significant correlation between the CoP and bow displacement (Sarro et al., 2021).

Furthermore, static unipedal balance correlates with putt distance after a chip shot and greens in regulation in elite golfers, suggesting that standing on uneven ground and weight shift during the golf swing may require good postural balance control (Wells et al., 2009). Unilateral balance also correlates with pitch velocity but not with pitching error in baseball players (Marsh et al., 2004).

An investigation of associations of balance with tennis expertise and performance revealed a lack of correlations, suggesting that postural stability measured in non-specific conditions is not a key factor of performance in the tennis serve (Caballero et al., 2021). However, sport experience in expert tennis players leads to better ability to perform postural adjustments (Caballero et al., 2021). A non-linear analysis is able to identify small postural adaptations induced by sport practice while the CoP dynamics discriminates sport expertise (Caballero et al., 2021).

With regard to dynamic balance, it is associated with maximum skating speed in young ice hockey players (Behm et al., 2005). Unipedal dynamic balance also correlates with starting speed during a simulated luge start in snowboarders (Platzer et al., 2009b), but not, however, with their ranking points (Platzer et al., 2009a).

### 3.3. A summary of studies published so far, their gaps and proposals for future research

While most of the analyzed studies reported an improvement of static and/or dynamic balance as a result of training including a variety of sport-specific balance exercises, a direct relationship between postural sway variables and athletic performance measures has been investigated and demonstrated only in few of them.

A relationship between static bipedal balance and shooting accuracy in rifle shooters was found (Ball et al., 2003) but only at the inter-individual level (Mononen et al., 2007). Postural stability correlates negatively with aiming accuracy and shooting score and positively with triggering and holding stability (Lang and Zhou, 2022a). In particular, aiming and holding are essential factors of shooting performance, followed by stability of triggering (Lang and Zhou, 2022b). Similarly, Ihalainen et al. (2016a) identified the aiming accuracy, cleanness of triggering, timing of triggering, and stability of hold as key predictors of shooting performance, whereas the effect of postural balance on performance is small (Ihalainen et al., 2016a). While postural balance control in cross-shooting direction is related to stability of hold, in shooting direction it is related to cleanness of triggering (Ihalainen et al., 2016b). Postural sway displacement prior to the arrow release is also associated with shooting performance in juniors but not in more experienced senior archers (Mason and Pelgrim, 1986). A significant correlation also exists between postural and bow stability in recurve archers (Sarro et al., 2021). Static unipedal balance correlates with criterion variables of performance in elite golfers (Wells et al., 2009). Unilateral stance is associated with pitch velocity but not with pitching error in baseball players (Marsh et al., 2004). Dynamic balance is related to maximum skating speed in young ice-hockey athletes (Behm et al., 2005). Dynamic unipedal balance is also associated with maximum starting speed during a luge start (Platzer et al., 2009b), however not with ranking points (Platzer et al., 2009a) in snowboarders. Although balance measured in non-specific conditions is not a key factor of performance in the tennis serve, sport experience in expert tennis players leads to specific adaptations demonstrated by better ability to perform proper postural adjustments (Caballero et al., 2021).

Taking these findings into account, it is clear that little attention has been paid to the relationship between postural sway variables and athletic performance measures in the existing research. Though balance exercises performed within a given sport may contribute to the improvement of neuromuscular control of postural stability, the transfer to sport-specific performance has not been sufficiently demonstrated. Therefore, further studies including general body conditioning exercises and specific exercises within particular sports and their applications for enhancing athletic performance should be conducted. Specifically, the effectiveness of various exercise programs in generalizing transfer to sport-specific skills should be evaluated. Neurophysiological mechanisms underpinning adaptive changes in postural balance control following these exercises should be more precisely addressed. A better understanding of long-term changes in body balance under conditions specific to particular sports and their associations with athletic performance can provide useful information for designing exercise programs best suited to individual athlete needs.

## 4. Conclusion

Out of 126 articles, only 14 investigated the association between variables of postural balance control and measures of sport-specific performance. A relationship between static and/or dynamic balance and criterion measures of athletic performance was revealed in shooting, archery, golf, baseball, ice-hockey, tennis, and snowboarding. This may be ascribed to improved ability of athletes to perform postural adjustments in highly balanced task demands. However, the extent to which sport-specific exercises contribute to their better postural stability is unknown. Although there is a good deal of evidence supporting neurophysiological adaptations in postural balance control induced by body conditioning exercises, little effort has been made to explain balance adaptations induced by sport-specific exercises and their effects on athletic performance. While an enhancement in the athletic performance is often attributed to an improvement of neuromuscular functions induced by sport-specific balance exercises, it can be equally well ascribed to their improvement by general body conditioning exercises. Therefore, the relevant experiments have yet to be conducted to investigate the relative contributions of each of these exercises to improving athletic performance.

## Author contributions

Both authors have made substantial, direct, and intellectual contribution to the work, and approved it for publication.

## References

[B1] Aalto H. Pyykkö I. Ilmarinen R. Kahkonen E. Starck J. (1990). Postural stability in shooters. *ORL J. Otorhinolaryngol. Relat. Spec.* 52 232–238. 10.1159/000276141 2392286

[B2] Agostini V. Chiaramello E. Canavese L. Bredariol C. Knaflitz M. (2013). Postural sway in volleyball players. *Hum. Mov. Sci.* 32 445–456. 10.1016/j.humov.2013.01.002 23628360

[B3] Andreeva A. Melnikov A. Skvortsov D. Akhmerova K. Vavaev A. Golov A. (2020). Postural stability in athletes: The role of age, sex, performance level, and athlete shoe features. *Sports (Basel)* 8:89. 10.3390/sports8060089 32560335 PMC7353649

[B4] Andreeva A. Melnikov A. Skvortsov D. Akhmerova K. Vavaev A. Golov A. (2021). Postural stability in athletes: The role of sport direction. *Gait Posture* 89 120–125. 10.1016/j.gaitpost.2021.07.005 34280882

[B5] Arksey H. O’Malley L. (2005). Scoping studies: Towards a methodological framework. *Int. J. Soc. Res. Methodol.* 8 19–32. 10.1080/1364557032000119616

[B6] Armstrong R. Hall B. J. Doyle J. Waters E. (2011). Cochrane update. ‘Scoping the scope’ of a cochrane review. *J. Public Health (Oxf)* 33 147–150. 10.1093/pubmed/fdr015 21345890

[B7] Asseman F. B. Caron O. Crémieux J. (2008). Are there specific conditions for which expertise in gymnastics could have an effect on postural control and performance? *Gait Posture* 27 76–81. 10.1016/j.gaitpost.2007.01.004 17337190

[B8] Asseman F. Caron O. Crémieux J. (2004). Is there a transfer of postural ability from specific to unspecific postures in elite gymnasts? *Neurosci. Lett.* 358 83–86. 10.1016/j.neulet.2003.12.102 15026154

[B9] Asseman F. Caron O. Crémieux J. (2005). Effects of the removal of vision on body sway during different postures in elite gymnasts. *Int. J. Sports Med.* 26 116–119. 10.1055/s-2004-830529 15726495

[B10] Aydin T. Yildiz Y. Yildiz C. Atesalp S. Kalyon T. A. (2002). Proprioception of the ankle: A comparison between female teenaged gymnasts and controls. *Foot Ankle Int.* 23 123–129. 10.1177/107110070202300208 11858332

[B11] Ball K. A. Best R. J. Wrigley T. V. (2003). Body sway, aim point fluctuation and performance in rifle shooters: Inter- and intra-individual analysis. *J. Sports Sci.* 21 559–566. 10.1080/0264041031000101881 12848390

[B12] Barbado Murillo D. Caballero Sánchez C. Moreside J. Vera-García F. J. Moreno F. J. (2017). Can the structure of motor variability predict learning rate? *J. Exp. Psychol. Hum. Percept. Perform.* 43 596–607. 10.1037/xhp0000303 28095006

[B13] Behm D. G. Muehlbauer T. Kibele A. Granacher U. (2015). Effects of strength training using unstable surfaces on strength, power and balance performance across the lifespan: A systematic review and meta-analysis. *Sports Med.* 45 1645–1669. 10.1007/s40279-015-0384-x 26359066 PMC4656700

[B14] Behm D. G. Wahl M. J. Button D. C. Power K. E. Kenneth G. Anderson K. G. (2005). Relationship between hockey skating speed and selected performance measures. *J. Strength Cond. Res.* 19 326–331. 10.1519/R-14043.1 15903370

[B15] Ben Moussa A. Z. Zouita S. Dziri C. Ben Salah F. Z. (2012). Postural control in Tunisian soccer players. *Sci. Sports* 27 54–56. 10.1016/j.scispo.2011.03.006

[B16] Borzucka D. Krêcisz K. Rektor Z. Kuczyński M. (2020a). Differences in static postural control between top level male volleyball players and non-athletes. *Sci. Rep.* 10:19334. 10.1038/s41598-020-76390-x 33168913 PMC7653955

[B17] Borzucka D. Krêcisz K. Rektor Z. Kuczyński M. (2020b). Postural control in top-level female volleyball players. *BMC Sports Sci. Med. Rehabil.* 12:65. 10.1186/s13102-020-00213-9 33101691 PMC7576872

[B18] Bressel E. Yonker J. C. Kras J. Heath E. M. (2007). Comparison of static and dynamic balance in female collegiate soccer, basketball, and gymnastics athletes. *J. Athl. Train.* 42 42–46. 17597942 PMC1896078

[B19] Bringoux L. Marin L. Nougier V. Barraud P. A. Raphel C. (2000). Effects of gymnastics expertise on the perception of body orientation in the pitch dimension. *J. Vestib. Res.* 10 251–258. 11455106

[B20] Busquets A. Ferrer-Uris B. Angulo-Barroso R. Federolf P. (2021). Gymnastics experience enhances the development of bipedal-stance multi-segmental coordination and control during proprioceptive reweighting. *Front. Psychol.* 12:661312. 10.3389/fpsyg.2021.661312 33935920 PMC8081832

[B21] Butler R. J. Bullock G. Arnold T. Plisky P. Queen R. (2016). Competition-level differences on the lower quarter Y-balance test in baseball players. *J. Athl. Train.* 51 997–1002. 10.4085/1062-6050-51.12.09 27849388 PMC5264565

[B22] Caballero C. Barbado D. Hérnandez-Davó H. Hernández-Davó J. L. Moreno F. J. (2021). Balance dynamics are related to age and levels of expertise. Application in young and adult tennis players. *PLoS One* 16:e0249941. 10.1371/journal.pone.0249941 33857225 PMC8049250

[B23] Caballero C. Barbado D. Urbán T. García-Herrero J. A. Moreno F. J. (2020). Functional variability in team-handball players during balance is revealed by non-linear measures and is related to age and expertise level. *Entropy (Basel)* 22:822. 10.3390/e22080822 33286592 PMC7517406

[B24] Calavalle A. R. Sisti D. Rocchi M. B. L. Panebianco R. Del Sal M. Stocchi V. (2008). Postural trials: Expertise in rhythmic gymnastics increases control in lateral directions. *Eur. J. Appl. Physiol.* 104 643–649. 10.1007/s00421-008-0815-6 18618136

[B25] Carrick F. R. Oggero E. Pagnacco G. Brock J. B. Arikan T. (2007). Posturographic testing and motor learning predictability in gymnasts. *Disabil. Rehabil.* 29 1881–1889. 10.1080/09638280601141335 17852265

[B26] Chapman D. W. Needham K. J. Allison G. Lay B. Edwards D. J. (2008). Effects of experience within a dynamic environment on postural control. *Br. J. Sports Med.* 42 16–21. 10.1136/bjsm.2006.033688 17496073

[B27] Chou T. Caccese J. B. Huang Y. Glutting J. J. Buckley T. A. Broglio S. P. (2022). Effects of pre-collegiate sport specialization on cognitive, postural, and psychological functions: Findings from the NCAA-DoD CARE consortium. *Int. J. Environ. Res. Public Health* 19:2335. 10.3390/ijerph19042335 35206522 PMC8871746

[B28] Croix G. Chollet D. Thouvarecq R. (2010). Effect of expertise level on the perceptual characteristics of gymnasts. *J. Strength Cond. Res.* 24 1458–1463. 10.1519/JSC.0b013e3181d2c216 20453683

[B29] Davlin C. D. (2004). Dynamic balance in high level athletes. *Percept. Mot. Skills* 98 1171–1176. 10.2466/pms.98.3c.1171-1176 15291203

[B30] Duchene Y. Mornieux G. Petel A. Perrin P. P. Gauchard G. C. (2021). The trunk’s contribution to postural control under challenging balance conditions. *Gait Posture* 84 102–107. 10.1016/j.gaitpost.2020.11.020 33290903

[B31] Edis Ç Vural F. Vurgun H. (2016). The importance of postural control in relation to technical abilities in small-sided soccer games. *J. Hum. Kinet.* 53 51–61. 10.1515/hukin-2016-0010 28149410 PMC5260576

[B32] Edis C. Vural F. Vurgun H. (2017). Does running performance in small-sided games have a relation with postural control in youth soccer players. *Turk. J. Sport Exerc.* 19 83–91.

[B33] Era P. Konttinen N. Mehto P. Saarela P. Lyytinen H. (1996). Postural stability and skilled performance: A study on top-level and naive rifle shooters. *J. Biomech.* 29 301–306. 10.1016/0021-9290(95)00066-6 8850636

[B34] Gerbino P. G. Griffin E. D. Zurakowski D. (2007). Comparison of standing balance between female collegiate dancers and soccer players. *Gait Posture* 26 501–507. 10.1016/j.gaitpost.2006.11.205 17197186

[B35] Glass S. M. Ross S. E. (2021). Direction-specific signatures of sport participation in center of pressure profiles of division I athletes. *Int. J. Sports Phys. Ther.* 16 1260–1272. 10.26603/001c.28227 34631246 PMC8486415

[B36] Glofcheskie G. O. Brown S. H. M. (2017). Athletic background is related to superior trunk proprioceptive ability, postural control, and neuromuscular responses to sudden perturbations. *Hum. Mov. Sci.* 52 74–83. 10.1016/j.humov.2017.01.009 28135584

[B37] Gómez-Landero L. A. Del Ojo P. L. Walker C. Floría P. (2021). Static balance performance differs depending on the test, age and specific role played in acrobatic gymnastics. *Gait Posture* 90 48–54. 10.1016/j.gaitpost.2021.07.023 34390922

[B38] González-Fernández F. T. Martínez-Aranda L. M. Falces-Prieto M. Nobari H. Clemente F. M. (2022). Exploring the Y-balance-test scores and inter-limb asymmetry in soccer players: Differences between competitive level and field positions. *BMC Sports Sci. Med. Rehabil.* 14:45. 10.1186/s13102-022-00438-w 35321733 PMC8944159

[B39] Granacher U. Behm D. G. (2022). Relevance and effectiveness of combined resistance and balance training to improve balance and muscular fitness in healthy youth and youth athletes: A scoping review. *Sports Med.* [Online ahead of print]. 10.1007/s40279-022-01789-7 36378414 PMC9876852

[B40] Halabchi F. Abbasian L. Mirshahi M. Mazaheri R. Shahi M. H. P. Mansournia M. A. (2020). Comparison of static and dynamic balance in male football and basketball players. *Foot Ankle Spec.* 13 228–235. 10.1177/1938640019850618 31122066

[B41] Hammami R. Chaouachi A. Makhlouf I. Granacher U. Behm D. G. (2016). Associations between balance and muscle strength, power performance in male youth athletes of different maturity status. *Pediatr. Exerc. Sci.* 28 521–534. 10.1123/pes.2015-0231 27046937

[B42] Han J. Waddington G. Anson J. Adams R. (2015). Level of competitive success achieved by elite athletes and multi-joint proprioceptive ability. *J. Sci. Med. Sport* 18 77–81. 10.1016/j.jsams.2013.11.013 24380847

[B43] Hrysomallis C. (2011). Balance ability and athletic performance. *Sports Med.* 41 221–232. 10.2165/11538560-000000000-00000 21395364

[B44] Ihalainen S. Kuitunen S. Mononen K. Linnamo V. (2016a). Determinants of elite-level air rifle shooting performance. *Scand. J. Med. Sci. Sports* 26 266–274. 10.1111/sms.12440 25850700

[B45] Ihalainen S. Linnamo V. Mononen K. Kuitunen S. (2016b). Relation of elite rifle shooters’ technique-test measures to competition performance. *Int. J. Sports Physiol. Perform.* 11 671–677. 10.1123/ijspp.2015-0211 26559498

[B46] Ihalainen S. Laaksonen M. S. Kuitunen S. Leppävuori A. Mikkola J. Lindinger S. J. (2018). Technical determinants of biathlon standing shooting performance before and after race simulation. *Scand. J. Med. Sci. Sports* 28 1700–1707. 10.1111/sms.13072 29446507

[B47] Jadczak Ł Grygorowicz M. Dzudziński W. Śliwowski R. (2019a). Comparison of static and dynamic balance at different levels of sport competition in professional and junior elite soccer players. *J. Strength Cond. Res.* 33 3384–3391. 10.1519/JSC.0000000000002476 29652679

[B48] Jadczak Ł Grygorowicz M. Wieczorek A. Śliwowski R. (2019b). Analysis of static balance performance and dynamic postural priority according to playing position in elite soccer players. *Gait Posture* 74 148–153. 10.1016/j.gaitpost.2019.09.008 31525652

[B49] Kiers K. Ellenberger L. Jermann J. Oberle F. Frey W. O. Spörri J. (2022). Prospective study on dynamic postural stability in youth competitive alpine skiers: Test-retest reliability and reference values as a function of sex, age and biological maturation. *Front. Physiol.* 13:804165. 10.3389/fphys.2022.804165 35480039 PMC9035548

[B50] Kim M. Kim Y. Kim H. Yoon B. (2018). Specific muscle synergies in national elite female ice hockey players in response to unexpected external perturbation. *J. Sports Sci.* 36 319–325. 10.1080/02640414.2017.1306090 28415899

[B51] Kioumourtzoglou E. Derri V. Mertzanidou O. Tzetzis G. (1997). Experience with perceptual and motor skills in rhythmic gymnasts. *Percept. Mot. Skills* 84 1363–1372. 10.2466/pms.1997.84.3c.1363 9229461

[B52] Kioumourtzoglou E. Derri V. Tzetzis G. Theodorakis Y. (1998). Cognitive perceptual, and motor abilities in skilled basketball performance. *Percept. Mot. Skills* 86 771–786. 10.2466/pms.1998.86.3.771 9656269

[B53] Ko J. H. Han D. W. Newell K. M. (2017). Skill level constrains the coordination of posture and upper-limb movement in a pistol-aiming task. *Hum. Mov. Sci.* 55 255–263. 10.1016/j.humov.2017.08.017 28858688

[B54] Ko J. H. Han D. W. Newell K. M. (2018). Skill level changes the coordination and variability of standing posture and movement in a pistol-aiming task. *J. Sports Sci.* 36 809–816. 10.1080/02640414.2017.1343490 28628398

[B55] Kodama K. Yamagiwa H. Yasuda K. (2021). Bimanual coordination in a whole-body dynamic balance sport, slacklining: A comparison of novice and expert. *Mot. Control* 25 462–474. 10.1123/mc.2020-0113 33992027

[B56] Konttinen N. Lyytinen H. Era P. (1999). Brain slow potentials and postural sway behaviour during sharpshooting performance. *J. Mot. Behav.* 31 11–20. 10.1080/00222899909601888 11177616

[B57] Konttinen N. Lyytinen H. Viitasalo J. (1998). Rifle-balancing in precision shooting: Behavioral aspects and psychophysiological implication. *Scand. J. Med. Sci. Sports* 8 78–83. 10.1111/j.1600-0838.1998.tb00172.x 9564711

[B58] Kozinc Ž Šarabon N. (2021). The effects of leg preference and leg dominance on static and dynamic balance performance in highly-trained tennis players. *PLoS One* 16:e0259854. 10.1371/journal.pone.0259854 34762690 PMC8584696

[B59] Lamoth C. J. van Lummel R. C. Beek P. J. (2009). Athletic skill level is reflected in body sway: A test case for accelometry in combination with stochastic dynamics. *Gait Posture* 29 546–551. 10.1016/j.gaitpost.2008.12.006 19138522

[B60] Lang D. Zhou A. (2022a). Relationships between postural balance, aiming technique and performance in elite rifle shooters. *Eur. J. Sport Sci.* 22 1493–1498. 10.1080/17461391.2021.1971775 34420487

[B61] Lang D. Zhou A. (2022b). Determinants of shooting performance in elite air rifle shooters. *Sports Biomech.* 1–11. [Online ahead of print]. 10.1080/14763141.2022.2055627 35343372

[B62] Larue J. Bard C. Otis L. Fleury M. (1989). Stability in shooting: The effect of expertise in the biathlon and in rifle shooting. *Can. J. Sport Sci.* 14 38–45. 2924221

[B63] Leightley D. Yap M. H. Coulson J. Piasecki M. Cameron J. Barnouin Y. (2017). Postural stability during standing balance and sit-to-stand in master athlete runners compared with nonathletic old and young adults. *J. Aging Phys. Act.* 25 345–350. 10.1123/japa.2016-0074 27768507

[B64] Lephart S. M. Smoliga J. M. Myers J. B. Sell T. C. Tsai Y. (2007). An eight-week golf-specific exercise program improves physical characteristics, swing mechanics, and golf performance in recreational golfers. *J. Strength Cond. Res.* 21 860–869. 10.1519/R-20606.1 17685707

[B65] Liang Y. Hiley M. Kanosue K. (2019). The effect of contact sport expertise on postural control. *PLoS One* 14:e0212334. 10.1371/journal.pone.0212334 30763383 PMC6375620

[B66] Makaracı Y. Soslu R. Özer Ö Uysal A. (2021). Center of pressure-based postural sway differences on parallel and single leg stance in Olympic deaf basketball and volleyball players. *J. Exerc. Rehabil.* 17 418–427. 10.12965/jer.2142558.279 35036391 PMC8743610

[B67] Marcolin G. Rizzato A. Zuanon J. Bosco G. Paoli A. (2019). Expertise level influences postural balance control in young gymnasts. *J. Sports Med. Phys. Fitness* 59 593–599. 10.23736/S0022-4707.18.08014-3 29845831

[B68] Marsh D. W. Richard L. A. Williams L. A. Lynch K. J. (2004). The relationship between balance and pitching error in college baseball pitchers. *J. Strength Cond. Res.* 18 441–446. 10.1519/R-13433.1 15320675

[B69] Mason B. R. Pelgrim P. P. (1986). Body stability and performance in archery. *Excel* 3 17–20.

[B70] Matsuda S. Demura S. Uchiyama M. (2008). Centre of pressure sway characteristics during static one-legged stance of athletes from different sports. *J. Sports Sci.* 26 775–779. 10.1080/02640410701824099 18409108

[B71] Michalska J. Kamieniarz A. Fredyk A. Bacik B. Juras G. Słomka K. J. (2018). Effect of expertise in ballet dance on static and functional balance. *Gait Posture* 64 68–74. 10.1016/j.gaitpost.2018.05.034 29879630

[B72] Michalska J. Zając R. Szydło K. Gerasimuk D. Słomka K. J. Juras G. (2022). Biathletes present repeating patterns of postural control to maintain their balance while shooting. *PLoS One* 17:e0267105. 10.1371/journal.pone.0267105 35503761 PMC9064080

[B73] Misegades J. Rasimowicz M. Cabrera J. Vaccaro K. Kenar T. DeLuccio J. (2020). Functional movement and dynamic balance in entry level university dancers. *Int. J. Sports Phys. Ther.* 15 548–556. 33354388 PMC7735684

[B74] Mocanu G. D. Murariu G. Onu I. Badicu G. (2022). The influence of gender and the specificity of sports activities on the performance of body balance for students of the faculty of physical education and sports. *Int. J. Environ. Res. Public Health* 19:7672. 10.3390/ijerph19137672 35805329 PMC9265780

[B75] Mohamed M. N. Azhar A. H. (2012). Postural sway and shooting accuracy of skilled recurve archers. *Mov. Health Exerc.* 1 49–60. 10.15282/mohe.v1i0.5

[B76] Mononen K. Konttinen N. Viitasalo J. Era P. (2007). Relationship between postural balance, rifle stability and shooting accuracy among novice rifle shooters. *Scand. J. Med. Sci. Sports* 17 180–185. 10.1111/j.1600-0838.2006.00549.x 17394480

[B77] Munzert J. Müller J. Joch M. Reiser M. (2019). Specificity of postural control: Comparing expert and intermediate dancers. *J. Mot. Behav.* 51 259–271. 10.1080/00222895.2018.1468310 29791278

[B78] Musa R. M. Abdulla M. R. Juahir H. Maliki A. B. H. M. Mat-Rasid S. M. Kosni N. A. (2018). A multidimensional analysis of physiological and mechanical variables among archers of different levels of expertise. *J. Fundam. Appl. Sci.* 10 18–32. 10.4314/jfas.v10i1s.2

[B79] Niinimaa V. McAvoy T. (1983). Influence of exercise on body sway in standing rifle shooting position. *Can. J. Appl. Sport Sci.* 8 30–33. 6850974

[B80] Nikolaidou M. Karfis V. Koutsouba M. Schroll A. Arampatzis A. (2021). Postural balance ability and the effect of visual restriction on older dancers and non-dancers. *Front. Sports Act. Living* 3:707567. 10.3389/fspor.2021.707567 34632376 PMC8494947

[B81] Noé F. Paillard T. (2005). Is postural control affected by expertise in alpine skiing? *Br. J. Sports Med.* 39 835–837. 10.1136/bjsm.2005.018127 16244193 PMC1725069

[B82] Olivier A. Viseu J. Vignais N. Vuillerme N. (2019). Balance control during stance–A comparison between horseback riding athletes and non-athletes. *PLoS One* 14:e0211834. 10.1371/journal.pone.0211834 30721260 PMC6363218

[B83] Omorczyk J. Bujas P. Puszczałowska-Lizis E. Biskup L. (2018). Balance in handstand and postural stability in standing position in athletes practicing gymnastics. *Acta Bioeng. Biomech.* 20 139–147. 30220715

[B84] Onofrei R. R. Amaricai E. (2022). Postural balance in relation with vision and physical activity in healthy young adults. *Int. J. Environ. Res. Public Health* 19:5021. 10.3390/ijerph19095021 35564412 PMC9105214

[B85] Opala-Berdzik A. Głowacka M. Juras G. (2021). Postural sway in young female artistic and acrobatic gymnasts according to training experience and anthropometric characteristics. *BMC Sports Sci. Med. Rehabil.* 13:11. 10.1186/s13102-021-00236-w 33579356 PMC7881629

[B86] Ozmen T. (2016). Relationship between core stability, dynamic balance and jumping performance in soccer players. *Turk. J. Sport Exerc.* 8 110–113. 10.15314/tjse.93545

[B87] Paillard T. (2017). Plasticity of the postural function to sport and/or motor experience. *Neurosci. Biobehav. Rev.* 72 129–152. 10.1016/j.neubiorev.2016.11.015 27894829

[B88] Paillard T. (2019). Relationship between sport expertise and postural skills. *Front. Psychol.* 10:1428. 10.3389/fpsyg.2019.01428 31293483 PMC6603331

[B89] Paillard T. Noé F. (2006). Effect of expertise and visual contribution on postural control in soccer. *Scand. J. Med. Sci. Sports* 16 345–348. 10.1111/j.1600-0838.2005.00502.x 16978254

[B90] Paillard T. Montoya R. Dupui P. (2007b). Postural adaptations specific to preferred throwing techniques practiced by competition-level judoists. *J. Electromyogr. Kinesiol.* 17 241–244. 10.1016/j.jelekin.2006.01.006 16563801

[B91] Paillard T. Bizid R. Dupui P. (2007a). Do sensorial manipulations affect subjects differently depending on their postural abilities? *Br. J. Sports Med.* 41 435–438. 10.1136/bjsm.2006.032904 17311808 PMC2465353

[B92] Paillard T. Costes-Salon C. Lafont C. Dupui P. (2002). Are there differences in postural regulation according to the level of competition in judoists? *Br. J. Sports Med.* 36 304–305. 10.1136/bjsm.36.4.304 12145123 PMC1724534

[B93] Paillard T. Margnes E. Portet M. Breucq A. (2011). Postural ability reflects the athletic skill level of surfers. *Eur. J. Appl. Physiol.* 111 1619–1623. 10.1007/s00421-010-1782-2 21193925

[B94] Paillard T. Noé F. Rivière T. Marion V. Montoya R. Dupui P. (2006). Postural performance and strategy in the unipedal stance of soccer players at different levels of competition. *J. Athl. Train.* 41 172–176. 16791302 PMC1472651

[B95] Pau M. Arippa F. Leban B. Corona F. Ibba G. Todde F. (2015). Relationship between static and dynamic balance abilities in Italian professional and youth league soccer players. *Phys. Ther. Sport* 16 236–241. 10.1016/j.ptsp.2014.12.003 25869425

[B96] Pau M. Porta M. Arippa F. Pilloni G. Sorrentino M. Carta M. (2018). Dynamic postural stability, is associated with competitive level, in youth league soccer players. *Phys. Ther. Sports* 35 36–41. 10.1016/j.ptsp.2018.11.002 30419410

[B97] Perrin P. P. Perrin C. A. Courant P. Béné M. C. Durupt D. (1991). Posture in basketball players. *Acta Otorhinolaryngol. Belg.* 45 341–347. 1950556

[B98] Perrin P. Deviterne D. Hugel F. Perrot C. (2002). Judo, better than dance, develops sensorimotor adaptabilities involved in balance control. *Gait Posture* 15 187–194. 10.1016/s0966-6362(01)00149-7 11869913

[B99] Platzer H. Raschner C. Patterson C. Lembert S. (2009a). Comparison of physical characteristics and performance among elite snowboarders. *J. Strength Cond. Res.* 23 1427–1432. 10.1519/JSC.0b013e3181aa1d9f 19620923

[B100] Platzer H. Raschner C. Patterson C. (2009b). Performance-determining physiological factors in the luge start. *J. Sports Sci.* 27 221–226. 10.1080/02640410802400799 19156559

[B101] Porter K. H. Quintana C. Morelli N. Heebner N. Winters J. Han D. Y. (2022). Neurocognitive function influences dynamic postural stability strategies in healthy collegiate athletes. *J. Sci. Med. Sport* 25 64–69. 10.1016/j.jsams.2021.07.012 34446366

[B102] Puszczałowska-Lizis E. Omorczyk J. (2019). The level of body balance in standing position and handstand in seniors athletes practicing artistic gymnastics. *Acta Bioeng. Biomech.* 21 37–44. 31741485

[B103] Rodrigues S. T. Polastri P. F. Gotardi G. C. Aguiar S. A. Mesaros M. R. Pestana M. B. (2016). Postural control during cascade ball juggling: Effects of expertise and base of support. *Percept. Mot. Skills* 123 279–294. 10.1177/0031512516660718 27502243

[B104] Rosker Z. M. Kristjansson E. Vodicar M. Rosker J. (2021). Postural balance and oculomotor control are influenced by neck kinaesthetic functions in elite ice hockey players. *Gait Posture* 85 145–150. 10.1016/j.gaitpost.2021.01.024 33578306

[B105] Sadowska D. Sacewicz T. Lichota M. Krzepota J. Ładyga M. (2019). Static postural balance in modern pentathletes: A pilot study. *Int. J. Environ. Res. Public Health* 16:1760. 10.3390/ijerph16101760 31109029 PMC6572282

[B106] Sarro K. J. Viana T. C. De Barros R. M. L. (2021). Relationship between bow stability and postural control in recurve archery. *Eur. J. Sport Sci.* 21 515–520. 10.1080/17461391.2020.1754471 32267203

[B107] Schmit J. M. Regis D. I. Riley M. A. (2005). Dynamic patterns of postural sway in ballet dancers and track athletes. *Exp. Brain Res.* 163 370–378. 10.1007/s00221-004-2185-6 15655686

[B108] Schram B. Hing W. Climstein M. (2016). Profiling the sport of stand-up paddle boarding. *J. Sports Sci.* 34 937–944. 10.1080/02640414.2015.1079331 26289320

[B109] Scinicarelli G. Offerhaus C. Feodoroff B. Froböse I. Wilke C. (2022). The association between multidirectional speed performance, dynamic balance and chronological age in young soccer players. *J. Funct. Morphol. Kinesiol.* 7:41. 10.3390/jfmk7020041 35736012 PMC9225006

[B110] Sell T. C. Tsai Y. Smoliga J. M. Myers J. B. Lephart S. M. (2007). Strength, flexibility, and balance characteristics of highly proficient golfers. *J. Strength Cond. Res.* 21 1166–1171. 10.1519/R-21826.1 18076270

[B111] Serrien B. Witterzeel E. Baeyens J.-P. (2018). The uncontrolled manifold concept reveals that the structure of postural control in recurve archery shooting is related to accuracy. *J. Funct. Morphol. Kinesiol.* 3:48. 10.3390/jfmk3030048 33466977 PMC7739306

[B112] Simsek D. Cerrah A. O. Ertan H. Soylu A. R. (2019). A comparison of the ground reaction forces of archers with different levels of expertise during the arrow shooting. *Sci. Sports* 34 e137–e145. 10.1016/j.scispo.2018.08.008

[B113] Śliwowski R. Marynowicz J. Jadczak Ł Grygorowicz M. Kalinowski P. Paillard T. (2021). The relationships between knee extensors/flexors strength and balance control in elite male soccer players. *PeerJ* 9:e12461. 10.7717/peerj.12461 34820190 PMC8603814

[B114] Snyder N. Cinelli M. (2020). Comparing balance control between soccer players and non-athletes during a dynamic lower limb reaching task. *Res. Q. Exerc. Sport* 91 166–171. 10.1080/02701367.2019.1649356 31479412

[B115] Sobera M. Serafin R. Rutkowska-Kucharska A. (2019). Stabilometric profile of handstand technique in male gymnasts. *Acta Bioeng. Biomech.* 21 63–71. 31197281

[B116] Spancken S. Steingrebe H. Stein T. (2021). Factors that influence performance in Olympic air-rifle and small-bore shooting: A systematic review. *PLoS One* 16:e0247353. 10.1371/journal.pone.0247353 33788853 PMC8011779

[B117] Spratford W. Campbell R. (2017). Postural stability, clicker reaction time and bow draw force predict performance in elite recurve archery. *Eur. J. Sport Sci.* 17 539–545. 10.1080/17461391.2017.1285963 28276913

[B118] Stambolieva K. Diafas V. Bachev V. Christova L. Gatev P. (2012). Postural stability of canoeing and kayaking young male athletes during quiet stance. *Eur. J. Appl. Physiol.* 112 1807–1815. 10.1007/s00421-011-2151-5 21909987

[B119] Stins J. F. Michielsen M. E. Roerdink M. Beek P. J. (2009). Sway regularity reflects attentional involvement in postural control: Effects of expertise, vision and cognition. *Gait Posture* 30 106–109. 10.1016/j.gaitpost.2009.04.001 19411174

[B120] Stuart J. Atha J. (1990). Postural consistency in skilled archers. *J. Sports Sci.* 8 223–234. 10.1080/02640419008732148 2084269

[B121] Sucharew H. Macaluso M. (2019). Methods for research evidence synthesis: The scoping review approach. *J. Hosp. Med.* 14 416–418. 10.12788/jhm.3248 31251164

[B122] Taube W. (2012). Neurophysiological adaptations in response to balance training. *Dtsch. Z. Sportmed.* 63 273–277. 10.5960/dzsm.2012.030

[B123] Taube W. Gruber M. Gollhofer A. (2008). Spinal and supraspinal adaptations associated with balance training and their functional relevance. *Acta Physiol. (Oxf)* 193 101–116. 10.1111/j.1748-1716.2008.01850.x 18346210

[B124] Thalassinos M. Fotiadis G. Arabatzi F. Isableu B. Hatzitaki V. (2018). Sport skill-specific expertise biases sensory integration for spatial referencing and postural control. *J. Mot. Behav.* 50 426–435. 10.1080/00222895.2017.1363704 28915093

[B125] Thorpe J. L. Ebersole K. T. (2008). Unilateral balance performance in female collegiate soccer athletes. *J. Strength Cond. Res.* 22 1429–1433. 10.1519/JSC.0b013e31818202db 18714247

[B126] Vendrame E. Belluscio V. Truppa L. Rum L. Lazich A. Bergamini E. (2022). Performance assessment in archery: A systematic review. *Sports Biomech.* 1–23. [Online ahead of print]. 10.1080/14763141.2022.2049357 35348423

[B127] Verhoeven F. M. Newell K. M. (2016). Coordination and control of posture and ball release in basketball free-throw shooting. *Hum. Mov. Sci.* 49 216–224. 10.1016/j.humov.2016.07.007 27442763

[B128] Vuillerme N. Danion F. Marin L. Boyadjian A. Prieur J. M. Weise I. (2001a). The effect of expertise in gymnastics on postural control. *Neurosci. Lett.* 303 83–86. 10.1016/s0304-3940(01)01722-0 11311498

[B129] Vuillerme N. Teasdale N. Nougier V. (2001b). The effect of expertise in gymnastics on proprioceptive sensory integration in human subjects. *Neurosci. Lett.* 311 73–76. 10.1016/s0304-3940(01)02147-4 11567781

[B130] Wada Y. Takeda N. (2020). Postural stability against full-field dynamic visual disturbance in archery players. *J. Med. Invest.* 67 67–69. 10.2152/jmi.67.67 32378620

[B131] Wang C. C. Yang W. H. (2012). Using detrended fluctuation analysis (DFA) to analyze whether vibratory insoles enhance balance stability for elderly fallers. *Arch. Gerontol. Geriatr.* 55 673–676. 10.1016/j.archger.2011.11.008 22169622

[B132] Wells G. D. Elmi M. Scott Thomas S. (2009). Physiological correlates of golf performance. *J. Strength Cond. Res.* 23 741–750. 10.1519/JSC.0b013e3181a07970 19387406

[B133] Wyatt H. E. Vicinanza D. Newell K. M. Irwin G. Williams G. K. R. (2021). Bidirectional causal control in the dynamics of handstand balance. *Sci. Rep.* 11:405. 10.1038/s41598-020-79730-z 33432011 PMC7801474

[B134] Yaggie J. A. Campbell B. M. (2006). Effects of balance training on selected skills. *J. Strength Cond. Res.* 20 422–428. 10.1519/R-17294.1 16686574

[B135] Zago M. Moorhead A. P. Bertozzi F. Sforza C. Tarabini M. Galli M. (2020). Maturity offset affects standing postural control in youth male soccer players. *J. Biomech.* 99:109523. 10.1016/j.jbiomech.2019.109523 31767282

[B136] Zemková E. (2010). “Sensorimotor exercises in sports training and rehabilitation,” in *Trends in human performance research*, eds Duncan M. J. Lyons M. (New York, NY: Nova Science Publishers Inc), 79–117.

[B137] Zemková E. (2014a). Significantly and practically meaningful differences in balance research: P values and/or effect sizes? *Sports Med.* 44 879–886. 10.1007/s40279-014-0185-7 24760590

[B138] Zemková E. (2014b). Sport-specific balance. *Sports Med.* 44 579–590. 10.1007/s40279-013-0130-1 24293269

[B139] Zemková E. (2022). Physiological mechanisms of exercise and its effects on postural sway: Does sport make a difference? *Front. Physiol.* 13:792875. 10.3389/fphys.2022.792875 35283801 PMC8908905

[B140] Zemková E. Hamar D. (2014). Physiological mechanisms of post-exercise balance impairment. *Sports Med.* 44 437–448. 10.1007/s40279-013-0129-7 24264058

[B141] Zemková, E. Zapletalová L. (2022). The role of neuromuscular control of postural and core stability in functional movement and athlete performance. *Front. Physiol.* 13:796097. 10.3389/fphys.2022.796097 35283763 PMC8909639

